# Word reading, vocabulary, and mental health problems in adolescent girls and boys with intellectual and developmental disabilities

**DOI:** 10.1080/20473869.2019.1626168

**Published:** 2019-06-18

**Authors:** Julia Eldblom, Petra Boström, Malin Broberg, Jakob Åsberg Johnels

**Affiliations:** 1 Habilitation & Health, Habilitation – Child and Youth, Region Västra Götaland, Gothenburg, Sweden;; 2 Speech and Language Pathology Unit & the Gillberg Neuropsychiatry Centre, Institute of Neuroscience and Physiology, University of Gothenburg, Gothenburg, Sweden;; 3 Department of Psychology, University of Gothenburg, Gothenburg, Sweden

**Keywords:** Intellectual and developmental disabilities, vocabulary, word reading, literacy, mental health problems

## Abstract

Reading difficulties are linked to several disadvantages in the general population. Less is known about correlates of reading difficulties in individuals with intellectual and severe developmental disabilities (IDD). Vocabulary and word reading were assessed in 112 adolescents with IDD, recruited from Special needs comprehensive schools in Sweden (*grundsärskolor* in Swedish). Proxy-ratings of mental health were collected from teachers and parents for a subset of the participants. Relationships between all measures were investigated. Reading and vocabulary were poorly developed in both groups and significantly associated. While mental health problems were common, there were no significant associations with word reading or with vocabulary knowledge. Thus, the study did not confirm an association between reading difficulties and mental health problems in adolescents with IDD. Still, the frequency of mental health problems and the low reading abilities point to the need for further intervention for adolescents with IDD.

Literacy can be defined as the ability to use reading and writing skills in everyday life (Vágvölgyi *et al.* 2016) and is highly significant for people’s participation, contribution, and well-being, as UNESCO highlights:

Literacy is a fundamental human right and the foundation for lifelong learning. It is fully essential to social and human development in its ability to transform lives. For individuals, families, and societies alike, it is an instrument of empowerment to improve one’s health, one’s income, and one’s relationship with the world.

In this study, we examined word reading – an important part of literacy – and vocabulary as well as their association with each other and with mental health in male and female adolescents with intellectual and severe developmental disabilities (IDD). These areas are related in the general population (e.g. Russell *et al.* 2015), but it is unknown whether this also applies to individuals with IDD.

The focus on word reading and vocabulary is motivated by the lexical quality hypothesis (Perfetti and Hart 2002), which states that knowledge of word forms and word meanings – typically assessed by word decoding and vocabulary tests, respectively – constitute central elements of an individual’s literacy facility. In support of the lexical quality model it has been shown that word decoding and vocabulary jointly predicts the development of reading comprehension in the general population (e.g. Horowitz-Kraus *et al.* 2017, Verhoeven *et al.* 2011).

### Reading difficulties and mental health problems in the general population

In the general population in Europe and the US, 5–12% of all children have reading difficulties (Horowitz-Kraus *et al.* 2017), with boys more often affected than girls (Hawke *et al.* 2009, Yoshimasu *et al*. 2010, but see Limbrick *et al.* 2012, for a counter example). Reading difficulties often co-occur with a broad range of other developmental and psychiatric difficulties (e.g. Cederlöf *et al.* 2017, Horowitz-Kraus *et al.* 2017). Importantly, several studies have shown a relationship between poor reading skills and mental health problems, including conduct problems and anxiety (e.g. Cederlöf *et al.* 2017, Carroll *et al.* 2005, Russell *et al.* 2015). In most studies, including ours, mental health problems are broadly defined, comprising both actual diagnoses, like anxiety disorder, and problematic behaviours that cause problems in everyday life, though not necessarily to a degree warranting a psychiatric diagnosis (Balazs *et al.* 2016). A broad definition enables the use of questionnaires to measure mental health problems, which is useful in research. In particular, the Strengths and Difficulties Questionnaire (SDQ, Goodman 1997) has been used for this purpose in a majority of the research which we refer to, including research of the general population, of poor readers and of groups with IDD.

The overall prevalence of mental health problems in children in the general population without IDD/DD is reported at around 13% (Kovess-Masfety *et al.* 2016, Stenmark *et al.* 2016). Boys often show a higher degree of mental health problems (e.g. Kovess-Masfety *et al.* 2016), though findings are mixed. In a Swedish study from 1999 (Smedje *et al.* 1999) boys in the general population had more problems with inattention and overall mental health, whereas girls had a higher degree of emotional problems. However, a more recent Swedish study found no pronounced gender differences (Stenmark *et al.* 2016).

### Reading difficulties, vocabulary, and mental health problems in individuals with IDD

Children with IDD often face reading difficulties (Ratz and Lenhard 2013, van Wingerden *et al.* 2017), and generally lag several years behind typically developed (TD) children in their reading development (e.g. Channell *et al*. 2013, van Wingerden *et al.* 2017). In a study of 1629 students with intellectual disability (ID) and a mean age of 13, Ratz and Lenhard (2013) found one third not being able to read at all, but the variation was considerable and one third read words fluently. There were no significant gender differences. IQ level and school grade accounted for the largest variance in reading level, with greater difficulties in students with a more severe ID (Ratz and Lenhard 2013).

Exploring the relationship between vocabulary and reading skills in children with ID, both van Tilborg *et al*. (2014) and van Wingerden *et al.* (2017) reported data that might be taken to suggest that vocabulary could be of less importance for both word reading and reading comprehension in children with ID, compared to TD children. Van Tilborg *et al.* (2014) suggest the vocabulary of the ID group might have been too poor to be pivotal for their word reading. In contrast to these findings, a longitudinal study showed oral vocabulary as a stronger predictor of the reading development for children with Down Syndrome (DS), compared to TD children (Hulme *et al.* 2012), and a meta-analytic review found that differences in vocabulary predicted variances in reading of non-words in children with DS (Naess *et al.* 2012). In sum, results regarding the association between oral vocabulary and word reading in children with IDD have been mixed and further studies are needed in order to shed light on the association between these critical skills (cf., Perfetti and Hart 2002).

Mental health problems, using the broad definition, are commonly reported in children with IDD attending special schools (Bakare *et al.* 2010, Cormack *et al.* 2000, Kaptein *et al.* 2008). Some studies have shown less pronounced gender differences than in the general population of children without IDD (Einfeld *et al.* 2010), while others indicate that boys have more difficulties (Bakare *et al.* 2010, Kaptein *et al.* 2008). In a literature review (Witwer and Lecavalier 2008), six of eleven studies showed gender differences, all with boys having significantly more externalising problems, while five did not find any significant gender differences.

### The present study

While the relationship between reading and mental health problems has been thoroughly explored in the general population, this is not the case for the IDD group. To our knowledge, there are in fact no such previous studies. The main aim of this study was therefore to explore word reading and its associations with oral vocabulary and mental health in adolescents with IDD. Secondary aims were to describe the word reading skills and vocabulary capacities of the participants and to describe the prevalence of mental health problems according to parent and teacher ratings. Comparisons between boys and girls were also carried out.

## Method

The data used in this study were collected in a larger project; the development of the Well-being in Special Education Questionnaire (WellSEQ; Boström *et al*. 2016).

### Participants

Special needs comprehensive schools (*grundsärskolan* in Swedish) in urban areas of Västra Götaland County, Sweden, that provided special education for adolescents with IDD, were contacted. Out of 33 schools, 18 accepted the invitation, and 113 (59%) of 190 students with IDD agreed to participate. One participant later withdrew, leaving 112 adolescents eligible for the current study. Participants (Table 1) were attending grades 5–10, had a mean age of 14 years, and 71% were boys. This gender ratio reflects the gender distribution of the population with IDD (Emerson 2003) but is slightly higher than in the publically available statistics on Special needs comprehensive schools in Västra Götaland, where the prevalence of boys is slightly above 60% (Skolverket 2014). The only inclusion criterion was that the adolescent was enrolled in a Special needs comprehensive school. The level of the ID and co-occurring diagnoses could neither be assessed nor retrieved from medical charts in this study (the ethical approval did not allow access to the participant’s medical charts). Nevertheless, we believe that the sample – which is heterogeneous – is representative of the constitution of classes in the Special needs comprehensive schools of Sweden.

**Table 1.** Gender and age of the participants (*n* = 112).

Gender	*n* (%)
Girls	32 (28.6)
Boys	80 (71.4)
Mean age	years (SD)
All (*n* = 110)	14.3 (1.4)
Girls (*n* = 30)	14.5 (1.3)
Boys (*n* = 80)	14.2 (1.4)
Age range	years
All (*n* = 110)	11–17
Girls (*n* = 30)	12–17
Boys (*n* = 80)	11–17

Parents and teachers of the participating adolescents were asked to fill out a web survey, including a proxy-rating of the adolescent’s mental health using the SDQ. This resulted in 67 (60%) and 94 (84%) of the adolescents being proxy-rated by their parents and teachers, respectively. Out of these, 60 adolescents (54% of the total sample) were rated by both their parents and teachers.

Sociodemographic and medical information, including the adolescents’ diagnoses was collected from parents via the web survey. Response rates to these questions were low, and therefore we only use data about the age and gender of the adolescents in this study. For those interested in the information that was given by the parents we refer to Boström *et al*. (2016).

## Procedure

### Ethical considerations

The procedure was approved by the regional ethical review board, ref. 417-13. Adolescents, parents and teachers obtained written information about the research process through letters. Adolescents and teachers also received oral information at their schools from a researcher or a research assistant. Participants were informed that they would receive a symbolic gift (€10 value) following participation, and that participation was voluntary and could be withdrawn at any time. Parents gave written consent for each adolescent’s participation, and the adolescents gave oral consent at the time of testing.

### Data collection

Data were collected from students at their schools by two of the authors and a research assistant, meeting one adolescent at a time. Adolescents first answered the WellSEQ (Boström *et al*. 2016) via an iPad, then partook in a test measuring word reading, and finally a test measuring vocabulary. Total testing time ranged from 18–45 minutes.

The web survey was open for parents and teachers around the time that data were collected at the schools, and were completed at a place and time chosen by themselves. If needed, reminders were emailed twice.

## Measures

### Vocabulary

The vocabulary subtest of the fourth edition of the Wechsler Intelligence Scale for Children (WISC IV Wechsler 2003) was used to measure vocabulary. The child is asked to define words, for example ‘What is an island?’. Scores for each question range from 0–2, depending on the child’s answer. All subscales of WISC are constructed to be able to measure superior intelligence, thus, maximum scores are outside most individuals’ reach. The reliability of the vocabulary subscale is estimated at .95 according to the Swedish validation (Tideman 2007).

For descriptive and analytical purposes, raw scores were converted into scaled scores (with a normative score of 10, SD = 3) and age equivalents. Raw scores ranging from 0–16 are equalled as <6:2 years, or <74 months. There were 83 adolescents (75.5%) with a raw score of ≤16, placing them in a very broad age equivalent subgroup. Raw data were also used in the analyses since they showed greater variability.

### Reading ability

Word reading was measured using the word reading part of the Swedish *Test i Läsning och Stavning* (LäSt) (Test of reading and spelling; Elwér *et al.* 2009). Single words are presented on two sheets, starting with easy two-letter words, followed by more difficult multi-syllable words. Participants are instructed to read as many words aloud as possible during two 45 second periods. The raw score is the sum of correctly read words from both sheets. Theoretically, the maximum score is 200, but the test is constructed to hinder ceiling effects and very few will reach this level. Reliability and validity of this instrument is reported in the manual (Elwér *et al.* 2009), and is proven good.

The LäSt raw scores were converted into Swedish school grade equivalents and age equivalents, again for descriptive and analytical purposes. A limit for calculating the age equivalent is set at a raw score of 51, which is the expected result at 93 months, or 7:9 years. (Children typically start school at age 7 years in Sweden). Thirty-four adolescents (31%) scored between 0–50. This subgroup was given the age equivalent of 92 months, or 7:8 years, to enable an inclusion in the analysis. Since this meant a very rough age equivalent, raw data were also used in the analyses.

### Mental health problems

The strengths and difficulties questionnaire (SDQ; Goodman 1997) was used to measure mental health problems in the adolescents, proxy-rated separately by parents and teachers. The SDQ is a well-established instrument for measuring mental health problems across different population samples and has shown good psychometric properties (e.g. Kaptein *et al.* 2008, Kovess-Masfety *et al.* 2016). It consists of 25 items, divided into five subscales, containing five statements each, measuring different dimensions of mental health/problems: prosocial behaviour, emotional symptoms, conduct problems, hyperactivity/inattention and peer problems. A three-point Likert-type scale is used, ranging from 0 (not true) to 2 (certainly true), with ratings based on the adolescent’s behaviour in the past six months. Each scale has a maximum score of 10. The prosocial scale reflects positive behaviours and a high score is expected from individuals with good mental health. The other four scales measure behavioural and emotional problems, and low scores are expected from individuals with good mental health. When added, the scores of these four scales constitute a total difficulties score, with a maximum score of 40. The total difficulties score can be used to screen for mental health problems, with a cut-off set at the 90^th^ percentile. In other words; 90% of the normative sample scores below the cut-off, and results above the cut-off reflect a high risk of clinically significant mental health problems (Youth in mind 2016). There are two Swedish validations of the SDQ for 5- to 15-year-old children rated by parents (Malmberg *et al.* 2003, Smedje *et al.* 1999), both reporting a mean total difficulties score of 6–7, and a cut-off at 14. There is no Swedish normative data for teacher ratings of school-aged children. Therefore, the British norms for teachers, with a cut-off at 16, were selected for this study. In our sample, the internal consistency of the scales was satisfactory: Cronbach’s alpha for the parent-rated SDQ scales ranged from .71 to .76, and the teacher-rated from .61 to .83.

### Missing data

Data were missing from two adolescents (1.8%) on the vocabulary test and from three (2.7%) on the word reading test. Missing values on the SDQ subscales were imputed according to instructions on the SDQ website (Youth in mind 2016).

### Data analysis

Mean raw scores were calculated for each measure. Raw scores of WISC-IV and LäSt were converted for each participant into age and grade equivalents, and scaled scores, following instructions in the test manuals. Since data were not normally distributed we used non-parametric tests to compare groups (boys vs girls; and subgroups based on ratings of mental health) and to conduct correlation analyses. Also chi-square test for independent samples was used when comparing girls and boys below/above the cut-off score in SDQ. SPSS versions 22 and 24 were used for all statistical calculations.

## Results

### Vocabulary and word reading

Means, standard deviations, and ranges of raw scores as well as converted scores on WISC-IV vocabulary and LäSt word reading are reported in Table 2. Compared to age norms the group scored poorly on both tests and several years below age expectations. The vocabulary corresponds to a mean scaled score of 1.7, and a mean age equivalent of 80 months, i.e. 6:8 years. The word reading score corresponds to the expected result in the second grade in Sweden, with a mean age equivalent of 106 months, i.e. 8:10 years.

**Table 2.** Group performances on the vocabulary subscale of WISC-IV and LäSt word reading, with means, standard deviations (SD), and ranges.

	Mean	SD	Range
WISC-IV vocabulary raw score^a^	14.3	7.8	0–38
WISC-IV vocabulary age equivalent^a^	80 months	15 months	73–146 months
WISC-IV vocabulary scaled score^b^	1.7	1.6	1–9
LäSt word reading raw score^c^	73.3	43.7	0–169
LäSt word reading age equivalent^c^	106 months	17 months	92–156 months
LäSt Word reading grade equivalent^c^	2.10	1.8	0–7

*Note*. The number of participants:

^a^ 110,

^b^ 108, and

^c^ 109.

### Mental health problems

The mean total difficulties score on SDQ was 14.4 (SD = 6.6, *n* = 67) when rated by parents and 10.0 (SD = 6.0, *n* = 94) when rated by teachers. There was a moderate, positive correlation between the parent- and teacher-rated total difficulties scores, *rho* = .45, *n* = 60, *p* < .001. Mean scores and standard deviations on each of the subscales are available in Table 3. According to parent ratings, 50.7% (*n* = 34) of the adolescents scored at or above the 90th percentile cut-off, i.e. had a total difficulties score of at least 14. According to teacher ratings, 19.1% (*n* = 18) of the adolescents scored at or above cut-off, i.e. had a total difficulties score of at least 16.

**Table 3.** Group means and standard deviations on the SDQ subscales, rated by parents and teachers.

	Parents (*n* = 67)	Teachers (*n* = 94)
Prosocial	7.4 (2.1)	6.7 (2.7)
Emotional symptoms	3.2 (2.3)	1.9 (1.7)
Conduct problems	2.3 (2.1)	1.6 (2.1)
Hyperactivity/inattention	5.1 (2.4)	4.0 (2.7)
Peer problems	3.8 (2.4)	2.5 (2.3)

### Gender differences in vocabulary and word reading

The only statistically significant difference between girls and boys was found on the scaled score of vocabulary, with a small-to-moderate effect (*p* = .0081, *rho* = .26) showing that girls performed lower. When controlling for multiple comparisons, using a Bonferroni adjusted alpha level of .0083, the correlation remained significant. Details of performances for and differences between girls and boys on the vocabulary subscale of WISC-IV and LäSt word reading are found in Table 4.

**Table 4.** Vocabulary subscale of WISC-IV and LäSt word reading means, standard deviations (SD), ranges, and group comparisons between girls and boys.

	Girls		Boys				
	Mean (SD)	Range	Mean (SD)	Range	*p*	*U*	*Z*
Vocabulary raw^a^	12.2 (5.3)	4–27	15.2 (8.5)	0–38	.10	999	−1.65
Vocabulary age^a^	76 (7)	73–102	82 (17)	73–146	.10	1061	−1.63
Vocabulary scaled^b^	1.13 (0.4)	1–3	1.97 (1.8)	1–9	<.01**	866	−2.65
LäSt reading raw^c^	74.0 (36.7)	2–154	73.0 (46.6)	0–169	.94	1220	−0.08
LäSt reading age^c^	104 (15)	92–147	106 (18)	92–156	.97	1226	−0.04
LäSt reading grade^c^	2.09 (1.6)	0–6	2.10 (1.9)	0–7	.86	1206	−0.18

*Note.* **p < .01. Vocabulary was assessed with WISC-IV, and is expressed in raw scores, age equivalents, and scaled scores (M = 10, SD = 3), respectively. Reading was assessed with LÄST, and is expressed in raw scores, age equivalents, and grade equivalents, respectively. Number of participants:

^a^ Girls = 32, boys = 78,

^b^ Girls = 30, boys = 78, and

^c^ Girls = 32, boys = 77.

### Gender differences in mental health problems

Mean SDQ scores with standard deviations and comparisons between girls and boys can be seen in Tables 5 (parents’ ratings) and Tables 6 (teachers’ ratings). There were no statistically significant differences between girls and boys on any subscale of the SDQ, when tested with a Mann Whitney U test. According to parents, 59.1% (*n* = 13) of the girls and 46.7% (*n* = 21) of the boys scored above the 90th percentile cut-off. According to teachers, this was the case for 16.7% (*n* = 4) of the girls and 20.0% (*n* = 14) of the boys. Chi-square tests for independence (with Yates Continuity Correction) revealed that the differences were not significant, neither according to parent ratings, χ^2^ (1, *n* = 67) = 0.48, *p* = .49, *phi* = −.12, nor according to the teacher ratings, χ^2^ (1, *n* = 94) = 0.00, *p* = .95, *phi* = .04.

**Table 5.** SDQ means and standard deviations, rated by parents, with group comparisons between girls and boys.

	Girls (*n* = 22)	Boys (*n* = 45)	*p*	*U*	*Z*
Prosocial	7.5 (2.3)	7.4 (2.0)	.70	466	−0.39
Emotional symptoms	3.4 (2.4)	3.1 (2.3)	.71	468	−0.37
Conduct problems	2.8 (2.3)	2.0 (2.0)	.10	375	−1.63
Hyperactivity/inattention	5.6 (2.6)	4.9 (2.3)	.35	425	−0.94
Peer problems	3.8 (2.1)	3.8 (2.6)	.95	491	−0.06
Total difficulties	15.5 (6.0)	13.9 (6.9)	.30	418	−1.04

**Table 6.** SDQ means and standard deviations, rated by teachers, with group comparisons between girls and boys.

	Girls (*n* = 24)	Boys (*n* = 70)	*p*	*U*	*Z*
Prosocial	6.6 (2.8)	6.7 (2.7)	.87	821	−0.17
Emotional symptoms	1.8 (1.8)	1.9 (1.7)	.62	784	−0.50
Conduct problems	1.6 (2.1)	1.6 (2.0)	.77	809	−0.29
Hyperactivity/inattention	3.3 (2.3)	4.3 (2.8)	.12	664	−1.54
Peer problems	2.9 (2.2)	2.4 (2.4)	.25	710	−1.15
Total difficulties	9.6 (6.1)	10.1 (6.0)	.54	770	−0.61

### Relationships between vocabulary, word reading, and mental health

Since there was only one measure that reached statistical significance when comparing girls and boys (i.e. the scaled score of vocabulary), correlations were investigated using data from the full group.

### The relationship between age and all measurements

As can be seen in Table 7, there was a small, significant, positive correlation between the age of the participants and two other measures; the vocabulary raw score (*rho* = .20, *p* = .04) and the peer problems subscale of SDQ, rated by parents (*rho* = .25, *p* = .04). When controlling for multiple comparisons, using an alpha level of .0071, none of the results remained significant.

**Table 7.** Bivariate non-parametric correlations between age, WISC-IV vocabulary scores and LäSt word reading scores.

	1	2	3	4	5	6	7
1. Age	–	.20* (108)	.18 (108)	−.15 (108)	.18 (107)	.19 (107)	.19 (107)
2. Vocabulary, raw		–	.76** (110)	.69** (108)	.53** (108)	.51** (108)	.52** (108)
3. Vocabulary, age			–	.78** (108)	.40** (108)	.40** (108)	.39** (108)
4. Vocabulary, scaled				–	.36** (106)	.35** (106)	.33** (106)
5. LäSt reading.raw					–	.98** (109)	.98** (109)
6. LäSt, reading age						–	.97** (109)
7. LäSt reading grade							–

*Note*. Vocabulary was assessed with WISC-IV, and is expressed in raw scores, age equivalents, and scaled scores (M = 10, SD = 3), respectively. Reading was assessed with LÄST, and is expressed in raw scores, age equivalents, and grade equivalents, respectively. The number of participants are presented within the parenthesis.

*p < .05.

^**^ *p* < .01.

### Relationships within and between vocabulary and word reading

There were significant correlations between all of the vocabulary and word reading scores, with moderate to large effect sizes (Table 7). All correlations were positive and remained so after controlling for multiple comparisons, with the adjusted alpha level of .0071.

### Relationships between vocabulary and word reading, and mental health problems

The only significant correlation found between vocabulary and mental health problems was a small, positive correlation between the vocabulary raw score and the hyperactivity/inattention subscale of SDQ rated by parents (*rho* = .28, *p* = .03). As for word reading, the only significant correlation was found between the LäSt age equivalent score and, again, the hyperactivity/inattention subscale of SDQ rated by parents (*rho* = .25, *p* = .05). When correcting the *p* value for multiple comparisons, neither of the correlations remained significant. There were no significant relationships between mental health problems rated by teachers and vocabulary or word reading. See Table 8 for details of the correlates.

**Table 8.** Bivariate non-parametric correlations between age, WISC-IV vocabulary scores, LäSt word reading scores, and SDQ scores.

	SDQ subscales rated by parents						SDQ subscales rated by teachers					
	Proso	Emo	Con	Hyp	Peer	Tot	Proso	Emo	Con	Hyp	Peer	Tot
Age^a^	.01	−.21	−.07	.06	.25*	−.00	.07	−.13	.02	.07	.01	−.01
Vocabulary, raw^b^	−.17	.07	.18	.28*	.16	.21	.06	−.05	.10	−.09	−11	−.07
Vocabulary, age^b^	−.12	.06	.15	.17	.15	.16	−.07	−.03	.16	.02	−.02	.03
Vocabulary, scaled^b^	−.19	.15	.18	.15	.10	.19	−.07	.07	.10	−.01	.01	.06
LäSt, reading.raw^c^	−.04	−.06	.12	.22	−.07	.08	−.16	−.03	.04	.03	−.11	−.04
LäSt, reading age^c^	−.03	−.04	.15	.25*	−.07	.11	−16	−.03	.06	.05	−.10	−.02
LäSt, reading grade^c^	−.02	−.05	.13	.24	−.07	.10	−.18	−.04	.09	.05	−.12	−.03

*Note.* Vocabulary was assessed with WISC-IV, and is expressed in raw scores, age equivalents, and scaled scores (M = 10, SD = 3), respectively. Reading was assessed with LÄST, and is expressed in raw scores, age equivalents, and grade equivalents, respectively. *Prosoc*: Prosocial; *Emotion*: Emotional Symptoms; *Conduct*: Conduct Problems; *Hyper*: Hyperactivity/inattention; *Peer*: Peer Problems; *Tot*: Total difficulties. Number of participants:

^a^ 67 = ratings by parents, 94 = ratings by teachers,

^b^ 65 = ratings by parents, 92 = ratings by teachers, and

^c^ 66 = ratings by parents, 91 = ratings by teachers.

*p < .05.

## Discussion

In this study, adolescents with IDD (mean age of 14 years) scored poorly on vocabulary and word reading, with results equivalent to that expected in the lower grades of elementary school. Proxy-ratings by their teachers and, especially, by their parents, clearly point to an overrepresentation of mental health problems. There were no significant gender differences on word reading, whereas vocabulary was the one area where girls and boys differed significantly, with girls performing worse than boys. Finally, there were significant, moderate-to-large correlations between vocabulary and word reading, but no significant correlations between these abilities and mental health/problems.

According to the lexical quality hypothesis (Perfetti and Hart 2002), word decoding and vocabulary constitute central elements of an individual’s literacy facility. Our results add to previous findings, and clearly show that vocabulary is an important correlate of word reading in the IDD population. Several studies have found a relationship of varying strength between oral vocabulary and word reading in individuals with IDD (e.g. Hulme *et al*. 2012, Sermier Dessemontet and de Chambrier 2015, van Wingerden *et al.* 2017, but see van Tilborg *et al.* (2014), for an exception). The moderate to strong correlations in our sample (irrespective of which measure of reading and vocabulary that was considered) exceed the strength of several others. What distinguishes our study from the rest is the age of the participants – they were, on average, more than three years older than the second oldest group (in Hulme *et al.* 2012). Approaching the idea of van Tilborg *et al.* (2014), it could be that children with IDD need to be considerably older than TD children in order to develop a vocabulary that is sufficient enough to underpin their word reading process. It could also be that a stronger relationship is seen between word reading and vocabulary when the child has a deeper understanding of the spoken word. It is possible that our vocabulary test – which is based on definitional capacities – captures this deeper comprehension/knowledge component to a greater extent than an identification task does, which has typically been used in previous research. These possibilities should be explored in future research.

In the general population, reading difficulties are seen more often in boys (Hawke *et al*. 2009, Yoshimasu *et al.* 2010) and girls have frequently been reported to excel in verbal skills, though this has been questioned by some (e.g. Hyde 2016). As for mental health problems, Stenmark *et al.* (2016) recently found gender differences to be less pronounced compared to previous research, and they propose it could be a reflection of a relatively strong gender equality in the studied, Swedish, population. Our finding that girls and boys with IDD did not differ significantly in word reading replicates that of Ratz and Lenhard (2013). Numerically, girls in our study seemed to score higher on several of the SDQ scales, but none of the differences were significant. In terms of vocabulary, somewhat poorer performance was seen in girls compared with boys, which stands out as a potentially contrasting result from earlier studies of both the general population and IDD groups. However, it is in line with findings in research on other neurodevelopmental disorders showing that while such problems in general are less prevalent in girls, when girls are identified for difficulties they are often more severely impaired than boys (e.g. Dworzynski *et al.* 2012). In this context, a potential weakness in our study needs to be mentioned as the prevalence of girls in the study sample might have been less than in the population of students in Special Needs Comprehensive Schools in the region (see page 6 for details). Thus, the generalizability of the results on gender differences needs to be determined in future research.

According to proxy-ratings by parents, more than 50% of the adolescents had clinically significant mental health problems. Proxy-ratings by teachers did not reach the same level, but at >20% it is still markedly above the prevalence in the general population (Kovess-Masfety *et al*. 2016, Stenmark *et al.* 2016). The differences between parents and teachers may raise some concerns regarding the validity of the ratings. However, differing views seem to be the rule rather than exception, according to a review of multi-informant assessment by De Los Reyes *et al.* (2015). Plausible reasons are that the adolescents actually display different behaviours in different settings, and that parents and teachers have different frames of reference, affecting their rating (De Los Reyes *et al.* 2015). It is also known that teachers are less sensitive to internalizing symptoms than parents. In the original WellSEQ project (Boström *et al.* 2016), self-ratings by the adolescents were collected which showed a somewhat different pattern of mental well-being than according parents and teachers. However, poorer reading levels were related to inconsistent responding, preventing us from using self-reports in this study, which specifically aimed to shed light on the association between reading skills and mental health. Including the perspectives of the adolescents themselves would be an important direction for future research.

The lack of relationships between mental health problems and the reading ability in our sample contrasts findings in the general population (e.g. Carroll *et al.* 2005, Russell *et al.* 2015). Although a null result like ours is always hard to interpret, one might speculate on its reasons. We have not found any previous research that have investigated the correlates of reading and mental health in adolescents with IDD, making direct comparisons with other studies impossible. However, in a study by Cormack *et al.* (2000), the mental health of 123 children with ID was proxy-rated by their parents, and its relationships with different developmental and medical problems, briefly assessed by a paediatrician, investigated. Language ability was not significantly correlated with mental health problems, but a strong relationship was found with broadly defined physical disabilities. It is possible that our results also could be explained by more severe problems in other areas than poor vocabulary and literacy abilities. Another potential cause to the lack of association has to do with the sensitivity of the test instruments used. Indeed, given the finding that an important minority was not able to read at all, the lack of correlations with mental health might be an artefact due to restriction of range. We tried to circumvent this problem by capitalising also on raw scores in the analyses, where the variability was greater than on the age-equivalent and standard scores, and with statistical techniques that did not assume normally distributed data. Also, and importantly, the fact that reading scores and vocabulary significantly correlate with one other suggest that these measures were sufficiently sensitive for an associational study.

In studies of the general population, there are several suggestions for the relationship between the reading difficulties and mental health problems. Carroll *et al.* (2005) and Russell *et al.* (2015) believe that some of the behavioural and emotional problems found to correlate with reading difficulties in their samples may be caused by the children not being able to cope with the academic demands in their school settings. But why then, is the association not clear in our sample? A study by Elbro (2010) can partly be related to this issue; he showed that individuals with specific reading disabilities and a large discrepancy between their strong vocabulary and poor reading ability experienced their reading difficulties as hindering them in everyday life, whereas individuals with smaller discrepancies did not. Taken together, these results may point to part of the relationship between reading difficulties and mental health problems being caused by demands in the environment and an awareness in the individual of not being able to live up to these demands and/or to one’s own potential. Such self-awareness would require the capacity to compare and value the abilities of one-self with that of others. Perhaps the adolescents in our sample are not (yet/fully) aware of their own difficulties. Being in a Special needs comprehensive school also means demands and expectations of academic as well as adaptive performances and behaviours will be lower than in the general population. Thus, it is possible that the adolescents are not experiencing their poor reading abilities as a cause of stress or frustration. Future studies should confirm or reject this speculative hypothesis. Moreover, another important issue more generally relates to the role of school placement for mental health difficulties in adolescents with IDD. The extent to which children with IDD in specialised schools versus in regular mainstream school settings differ in their mental health outcomes is a contested and highly relevant topic for future research.

It should also be stressed that mental health is only one area of health that has been shown to correlate with the reading ability. In the general population, having poor literacy skills is linked to an increased risk of problems in several areas of life, including poverty, inadequate nutritional and health practices, a higher degree of high-risk sexual behaviours, less knowledge about, and therefore less possibility to claim, fundamental rights and lower self-esteem (Martinez and Fernandez 2010, Vágvölgyi *et al.* 2016). Thus, there are many other aspects of health and their possible relationship with the reading ability in individuals with IDD that remains to be investigated. Moreover, even though the SDQ was deemed suitable for this study since it is widely used and has been used in studies of several clinical populations, it is still only one measurement; indeed, we cannot rule out that other ways of measuring mental health might yield a different result. We still believe it is very likely that being literate as an adult will be of great significance for the wellbeing of the IDD population, as has been established for the general population (e.g. Martinez and Fernandez 2010).
